# FOXA1/MND1/TKT axis regulates gastric cancer progression and oxaliplatin sensitivity via PI3K/AKT signaling pathway

**DOI:** 10.1186/s12935-023-03077-4

**Published:** 2023-10-10

**Authors:** Xiaosi Hu, Shuai Zhou, Haohao Li, Zehui Wu, Ye Wang, Lei Meng, Zhangming Chen, Zhijian Wei, Qing Pang, Aman Xu

**Affiliations:** 1 Department of General Surgery, Anhui No.2 Provincial People’s Hospital, Hefei, 230041 Anhui People’s Republic of China; 2 Department of General Surgery, The First Affiliated Hospital of Anhui Medical University, Hefei, 230001 Anhui People’s Republic of China; 3 Department of General Surgery of Anhui Public Health Clinical Center, Hefei, 230001 Anhui People’s Republic of China

**Keywords:** Gastric cancer, Meiotic nuclear divisions 1, Forkhead box protein A1, Transketolase, PI3K/AKT signaling pathway

## Abstract

**Background:**

Drug resistance is a main factor affecting the chemotherapy efficacy of gastric cancer (GC), in which meiosis plays an important role. Therefore, it is urgent to explore the effect of meiosis related genes on chemotherapy resistance.

**Methods:**

The expression of meiotic nuclear divisions 1 (MND1) in GC was detected by using TCGA and clinical specimens. In vitro and in vivo assays were used to investigate the effects of MND1. The molecular mechanism was determined using luciferase reporter assay, CO-IP and mass spectrometry (MS).

**Results:**

Through bioinformatics, we found that MND1 was highly expressed in platinum-resistant samples. In vitro experiments showed that interference of MND1 significantly inhibited the progression of GC and increased the sensitivity to oxaliplatin. MND1 was significantly higher in 159 GC tissues in comparison with the matched adjacent normal tissues. In addition, overexpression of MND1 was associated with worse survival, advanced TNM stage, and lower pathological grade in patients with GC. Further investigation revealed that forkhead box protein A1 (FOXA1) directly binds to the promoter of MND1 to inhibit its transcription. CO-IP and MS assays showed that MND1 was coexpressed with transketolase (TKT). In addition,TKT activated the PI3K/AKT signaling axis and enhanced the glucose uptake and lactate production in GC cells.

**Conclusions:**

Our results confirm that FOXA1 inhibits the expression of MND1, which can directly bind to TKT to promote GC progression and reduce oxaliplatin sensitivity through the PI3K/AKT signaling pathway.

**Supplementary Information:**

The online version contains supplementary material available at 10.1186/s12935-023-03077-4.

## Introduction

Gastric cancer (GC) is one of the malignant tumors with high mortality [1]. The 5-year survival rate of GC is less than 20% due to the high incidence of metastasis [2–4]. Chemotherapeutic resistance after surgery is therefore a huge challenge in patients with middle and late stages of GC [5]. As one of the most common chemotherapeutic agents, oxaliplatin inhibits DNA synthesis and delays the progression of GC [6]. Overcoming oxaliplatin resistance is one of the key approaches in the adjuvant therapy of GC.

Deviations in the mechanisms of chromosome maintenance and segregation caused by inappropriate activation of meiotic chromosome regulatory genes are critical in chemotherapy resistance [6, 7]. We recently used bioinformatics to screen optimal genes in platinum-resistant tissues and found that meiotic nuclear divisions 1 (MND1) was significantly upregulated. As a meiotic regulatory protein, MND1 can form a complex with HOP2 to promote homologous chromosome pairing and meiotic double-strand break repair [8]. Meanwhile, the MND1-HOP2 complex can drive meiotic recombination into the interhomologous pathway, which replaces the telomere elongation mechanism in cancer cells [9]. MND1, Kruppel-like factor 6 (KLF6), and E2F transcription factor 1 (E2F1) can form a positive feedback loop to confer cisplatin resistance [10]. However, the role of MND1 in GC and oxaliplatin resistance remains unknown.

Glucose metabolism and chemotherapy resistance are inseparable in tumor progression [11]. Reprogramming of glucose metabolism after chemotherapy is a hallmark of cancer progression, especially with respect to an increase in aerobic glycolysis [12]. Transketolase (TKT) is involved in the pentose phosphate pathway and is upregulated in various cancers [13]. TKT regulates NADPH and EGFR pathway to promote liver cancer progression [14]. AKT phosphorylates and activates TKT activity [12], and in turn, the increase of TKT promotes the expression of AKT [12]. TKT promotes the resistance of colorectal cancer and breast cancer cells to oxaliplatin [15]. However, whether MND1 could regulate TKT and participate in the progression of GC remain unclear.

Herein, we demonstrated that, MND1 could regulate cell cycle, apoptosis, proliferation, metastasis and oxaliplatin resistance, and play an oncogene role in GC. Forkhead box protein A1 (FOXA1) could directly binds to the promoter of MND1 to inhibit its expression. Moreover, MND1 was observed to be coexpressed with TKT, which further promoted GC progression via activating PI3K/AKT signaling pathway. These results suggest that MND1 may be a potential therapeutic target in GC.

## Materials and methods

### Tissue collection

Six pairs of GC cancer tissues and adjacent normal tissues were collected at the First Affiliated Hospital of Anhui Medical University. All patients provided their signed informed consent. Our study received approval from the Ethics Committee of Anhui Medical University.

### Cell culture

Normal gastric epithelial cell GES-1, and GC cell lines MNK-45, SGC-7901, SGC-803, HGC27, and AGS were all purchased from the Cell Bank of the Chinese Academy of Sciences. Cells were cultured in the RPMI-1640 medium (Gibco, USA) supplemented with 10% fetal bovine serum (FBS; Gibco).

### RNA interference and plasmid construction

The sequences of *MND1*, *FOXA1*, and *TKT* were amplified by PCR, and the target sequences were subcloned into a pEGFP-N1 vector according to the restriction sites. The recombinant plasmids were amplified into monoclonal colonies (Solarbio, China).

The double-stranded DNA was digested with multiple enzymes and was inserted into the RNAi lentiviral pLENR-GPH vector. The LV-shMND1 sequence:5ʹ-CCGGCGCAAGTTGTGGAAGAAATTTCTCGAGAAATTTCTTCCACAACTTGCGTTTTTGAATTC-3ʹ and the Lv-shTKT sequence: 5ʹ-TCACCGTGGAGGACCATTATTCTCGAGAATAATGGTCCTCCACGGTGATTTTT-3ʹ. The ligated products were used to transform the competent cells of *E. coli*, and the recombinants were detected by PCR. Then, the recombinant lentivirus (100 μL, 1 × 10^7^ units/mL) containing green fluorescent protein (GFP) were cultured at the density of 2 × 10^5^ cells/well. At 24 h after transfection with the lipofectamine 3000 transfection reagent, the fluorescence intensity was determined.

### Bioinformatic analyses

The TPKM data of the STAD data were downloaded from the TCGA database (https://tcgadata.nci.nih.gov/tcga). After normalization, paired and unpaired analyses were performed to explore the expression differences of MND1 in the STAD data. The GEO database (https://www.ncbi.nlm.nih.gov/geo/) GSE70880 (https://www.ncbi.nlm.nih.gov/geo/query/acc.cgi?acc=GSE70880), GSE99416 (https://www.ncbi.nlm.nih.gov/geo/query/acc.cgi?acc=GSE99416) and GSE122130 (https://www.ncbi.nlm.nih.gov/geo/query/acc.cgi?acc=GSE122130) data sets were downloaded. Differential gene analysis was then performed using the "limma" R package.

The differential genes of each data set were shown in Additional file 2: Table S1 and Additional file 3: Table S2. Meanwhile, the GEPIA database (http://gepia.cancer-pku.cn/index.html) was used to detect the expression of MND1 in each type of cancer. hTFtarget (http://bioinfo.life.hust.edu.cn/hTFtarget#!/) and PROMO (http://alggen.lsi.upc.es/cgi-bin/promo_v3/promo/promoinit.cgi? dirDB = TF_8.3) database were employed to search for the transcription factors that bind to MND1.

### RNA extraction and qRT-PCR analysis

QRT-PCR was performed as previously described [16]. The Trizol lysis buffer (Tiangen, China) was used to extract the total cell RNA, which was further processed with the reverse transcription kit (Transgen, China) with the addition of the corresponding reagents mentioned by the manufacturer. The primers were shown in Additional file 4: Table S3. According to the requirements of the amplification kit, the necessary reagents were added to the 8-connected tubes for subsequent PCR analyses.

### Western blotting

WB was performed as previously described [16]. The samples were separated by gel electrophoresis SDS-PAGE, then transferred to the membrane. The membrane was washed thrice with TPBS for 30 min, blocked with blocking solution for 30 min and incubated with primary antibody (Additional file 5: Table S4). After overnight, the membrane was exposed to the ECL chemiluminescence reagent. Finally, the bands were analyzed with the Image J software.

### CCK-8 and cell cloning experiments

CCK-8 and cell cloning experiments were performed as previously described [16]. The cell density of 5000 cells/well was seeded in 96-well plate. CCK8 reagent was added to each well and the absorbance was measured at 450 nm. The cells in each group were seeded in a 6-well plate at the density of 1000 cells/well and cultured for 12 days. The pictures were taken after dyeing.

### EDU assay

The treated cells from each group were planted in a 12-well plate and incubated with EDU (Beyotime) solution. Then, the cells were washed thrice, treated with 4% paraformaldehyde, fixed, and re-washed thrice with a permeabilization solution. According to the instructions, the reaction solution was prepared and was added to the cells. The cells were then configured with the DAPI staining solution, and the images were taken under a fluorescence microscope.

### Cell cycle and apoptosis analysis

The cells in each group were digested, centrifuged, and fixed with 75% ethanol, and was further processed according to the cell cycle kit manufacturer’s instruction (Beyotime). Cell apoptosis was determined by using the Annexin V-APC/PI apoptosis kit (Biolite, China) and analyzed by using flow cytometry.

### Dual-luciferase reporter assay

The MND1 target sequence was subcloned into the pGL3-basic vector, and the FOXA1 target sequence was transferred into the pEGFP-N1 vector. The recombinant plasmid was used for monoclonal cloning and amplification. 293 T cells were co-transfected with the MND1 or FOXA overexpression vector, empty pGL3, pGL3 promoter plasmid, and pRL vector luciferase (internal control). Luciferase activity was detected after 24 h.

### Invasion, migration, and wound healing assays

The migration and invasion experiments were conducted as described previously [17]. Transwell chambers was used to detect migration and invasion. The confluent cells were scratched horizontally in the 6-well plate and pictures were taken at 0 and 48 h, respectively.

### Liquid chromatography-tandem mass spectrometry (LC–MS/MS) and co-immunoprecipitation (CO-IP)

The MND1-binding proteins were identified using HGC27 cells transfected with the Flag-MND1 vector. The cells were lysed by using the CO-IP kit (Absin, China) to obtain the input, IP, and IgG groups. The expressions of MND1 in the input, IP, and IgG groups were detected by WB. Subsequent LC–MS/MS analysis was performed by using a high-resolution mass spectrometer. CO-IP analysis was performed according to the obtained results.

### Lactate production and glucose uptake assays

The cells in each group were treated with tissue lysate, and were centrifuged at 12,000 ×*g* for 5 min. Then, the supernatant was collected. The operation was further performed according to the manufacturer instructions of the glucose detection kit (Beyotime), and the absorbance was measured at 630 nm. The glucose content in the culture medium was measured according to the standard curve. Lactic acid production was determined and normalized by using the L-lactic acid detection kit (Solarbio).

### Immunofluorescence (IF) staining

The medium was removed after the cells being treated in each group. Then, the cells were fixed with 4% paraformaldehyde, permeabilized with 0.1% Triton X 100, blocked with goat serum, and incubated with the primary antibodies (Additional file 5: Table S4). After overnight, the cells were washed thrice with PBS, incubated with a secondary antibody (Beyotime), and stained with DAPI for nuclei staining.

### Tissue microarray (TMA) and immunohistochemistry (IHC)

A total of 159 pairs of GC cancer tissues (gastric adenocarcinoma) and adjacent tissues were collected from the First Affiliated Hospital of Anhui Medical University to perform the microarray tissue chips. Clinical characteristics of the patients were shown in Additional file 6: Table S5. IHC was performed as previously described [16]. Antigen retrieval was performed and 3% hydrogen peroxide was used to block endogenous peroxidase. The slices were treated with the immunoblocking solution and were incubated with the primary antibody (Additional file 5: Table S4). Finally, the coverslips were air-dried and the slices were photographed under a fluorescence microscope.

### Animal study

Four-week-old BALB/c female nude mice were purchased from Shanghai Lingchang Biotechnology Co., Ltd., and the animal experiments were approved by the ethics of the Anhui Medical University. The HGC27 cells were stably transfected, digested, and centrifuged in each group at the logarithmic growth phase. The cells were re-spun with a mixture of 100 μL/tube and were inoculated into the axilla of mice (1 cm). The tumor volume and growth curve were observed and measured. Mice were anesthetized with 1% chloral hydrate. Then, the removed tumors were sorted in a descending order and were photographed. Finally, the mice were killed by de-necking, and the skin on the tumor body was peeled off with ophthalmic scissors.

### Statistical analyses

Biological analysis Use Rstudio V4.0.2 version was used to analyze the downloaded FPKM data. Statistical analysis was performed using Prism 8.0 (Graphpad software). Chi-square test was applied to compare the count data. The differences between the groups were analyzed by independent *t*-test or one-way analysis of variance (ANOVA). Overall survival (OS) was plotted using the Kaplan–Meier method and was analyzed using the COX regression model. *P* < 0.05 was considered to be statistically significant. All experiments were repeated thrice.

## Results

### The overexpression of MND1 was correlated with poor prognosis in GC patients

The differential genes were screened from TCGA, GSE70880 and GSE99416. Amongst, there were 80 intersection genes (Additional file 1: Fig. S1A). Subsequently, platinum resistance data set GSE122130 was used to further screen differential genes and 4 intersection genes (CENPU, CDH17, MND1, CDC45) were screened out (Additional file 1: Fig. S1A). Based on the GEPIA tool, MND1 was found to be overexpressed in various types of cancer (Additional file 1: Fig. S1B).

**Fig. 1:**
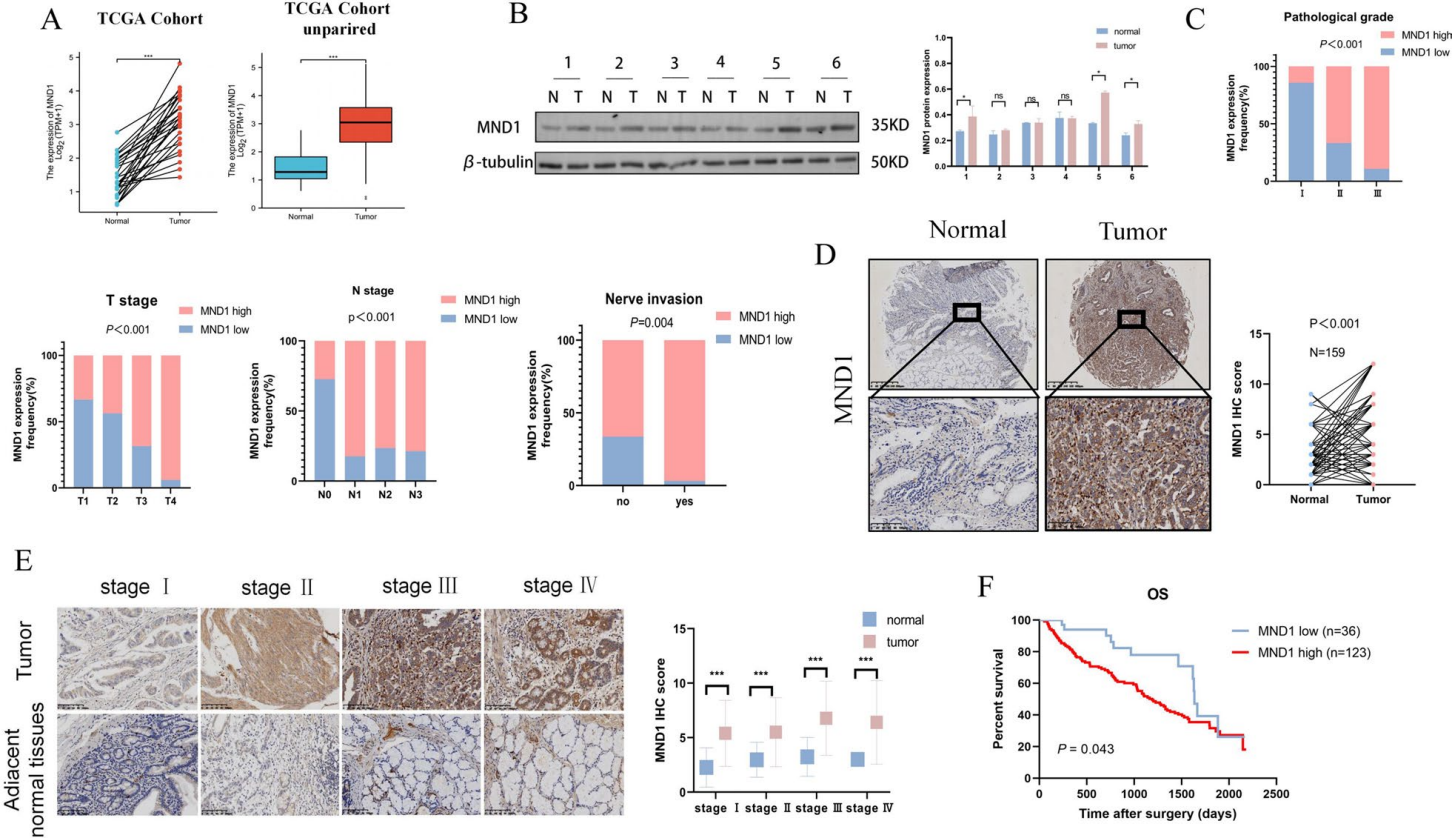
MND1 is overexpressed and is associated with poor prognosis in GC patients. **A**. MND1 was elevated in GC tissue compared with the normal tissue based on TCGA database. **B** MND1 expressions in 6 pairs of GC tissues and adjacent normal tissues were ananlyzed by WB. **C** Immunohistochemical analysis showed that MND1 was correlated with pathological stage, T stage, N stage and Nerve invasion in 159 GC patients. **D** Immunohistochemistry was used to detect the expression of MND1 in paired tumor tissues and adjacent normal tissues in 159 GC patients. **E** Immunohistochemistry was used to detect MND1 expression levels in paired tumor and adjacent normal tissues stratified according to different pathological stages. **F** The overall survival (OS) was evaluated by the Kaplan–Meier survival curve and the log-rank test from 159 GC patients according to MND1 expression levels (*P-value < 0.05, **P-value < 0.01, ***P-value < 0.001)

The baseline data of 159 GC patients were shown in Additional file 7: Table S6. According to the statistical results of the tissue microarray chip of 159 GC patients, high expression of MND1 was associated with a worse prognosis at TNM stage III (Additional file 1: Fig. S1C). Tissue microarray was shown in Additional file 1: Fig. S2. Based on the TCGA database and 6 pairs of GC patients, MND1 was significantly elevated in tumor tissue compared with normal tissue (Fig. 1A, B). The IHC results of 159 GC patients demonstrated that the higher expression of MND1 was significantly associated with advanced pathological grade, T stage, N stage, and Nerve invasion (Fig. 1C). The expression of MND1 were positively related to pathological stage (Fig. 1D, E). Patients with a high expression of MND1 had a worse OS (Fig. 1F). Furthermore, MND1 was indentified as an independent prognostic factor (Additional file 8: Table S7). The above results indicated that, MND1 was overexpressed in GC patients and was an unfavorable prognostic factor.

### MND1 silencing inhibited the proliferation of GC cells and caused cell cycle arrest in the G1 phase

**Fig. 2:**
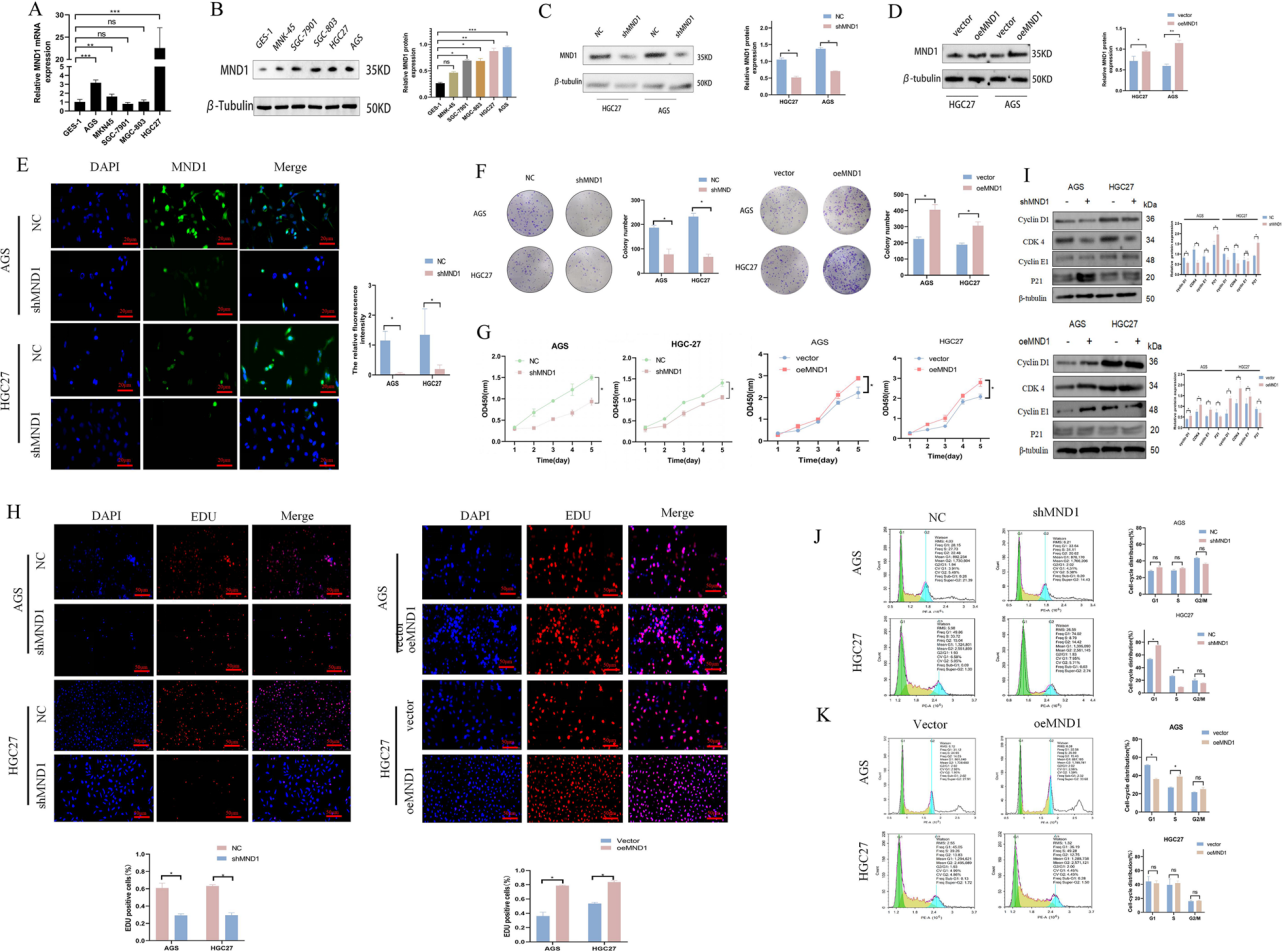
MND1 promotes the proliferation of GC cells and induces cell cycle arrest in the G1 phase. **A, B** QRT-PCR and WB were used to detect MND1 expression in GC cells. **C, D** WB was used to detect the levels of MND1 after silencing and overexpression in GC cells. **E** The localization and silencing efficiency of MND1 cells were detected by immunofluorescence assay. **F, G** Colony forming and CCK-8 assays were used to detect the proliferation after silencing and overexpression of MND1. **H** EDU was used to detect cell proliferation after silencing and overexpression of MND1. **I** The changes of cell cycle related protein expression in each group were detected by WB. **J, K** Cell cycle changes were detected by flow cytometry after silencing and overexpression of MND1. (*P-value < 0.05, **P-value < 0.01, ***P-value < 0.001). All figures represent mean ± SD from three independent experiments

Compared with GES-1 cell, expression of MND1 was increased in all GC cells, especially in HGC27 and AGS cells (Fig. 2A, B). Therefore, the two cell lines were further selected. We then constructed a vector with silenced or overexpressed MND1 and transfected it into AGS and HGC27 cells. WB confirmed that the transfection efficiency was good (Fig. 2C, D). Immunofluorescence results showed that MND1 was enriched in cytoplasm (Fig. 2E).

CCK-8, cell cloning and EDU experiments showed that the cell proliferation efficiency was significantly reduced in shMND1 group compared with NC group (Fig. 2F–H). In contrast, the overexpression of MND1 increased the proliferation of GC cells. After MND1 silencing, the number of cells arrested in G1 phase increased, the expression of P21 increased, and the expression of Cyclin D1, CDK4, and Cyclin E1 decreased (Fig. 2I, J). In contrast, overexpression of MND1 promoted the cell cycle progression of GC cells (Fig. 2 K). Taken together, MND1 silencing could inhibit the proliferation of GC cells and arrest the cell cycle in the G1 phase.

### MND1 silencing inhibited the invasion and migration of AGS and HGC27 cells

**Fig. 3:**
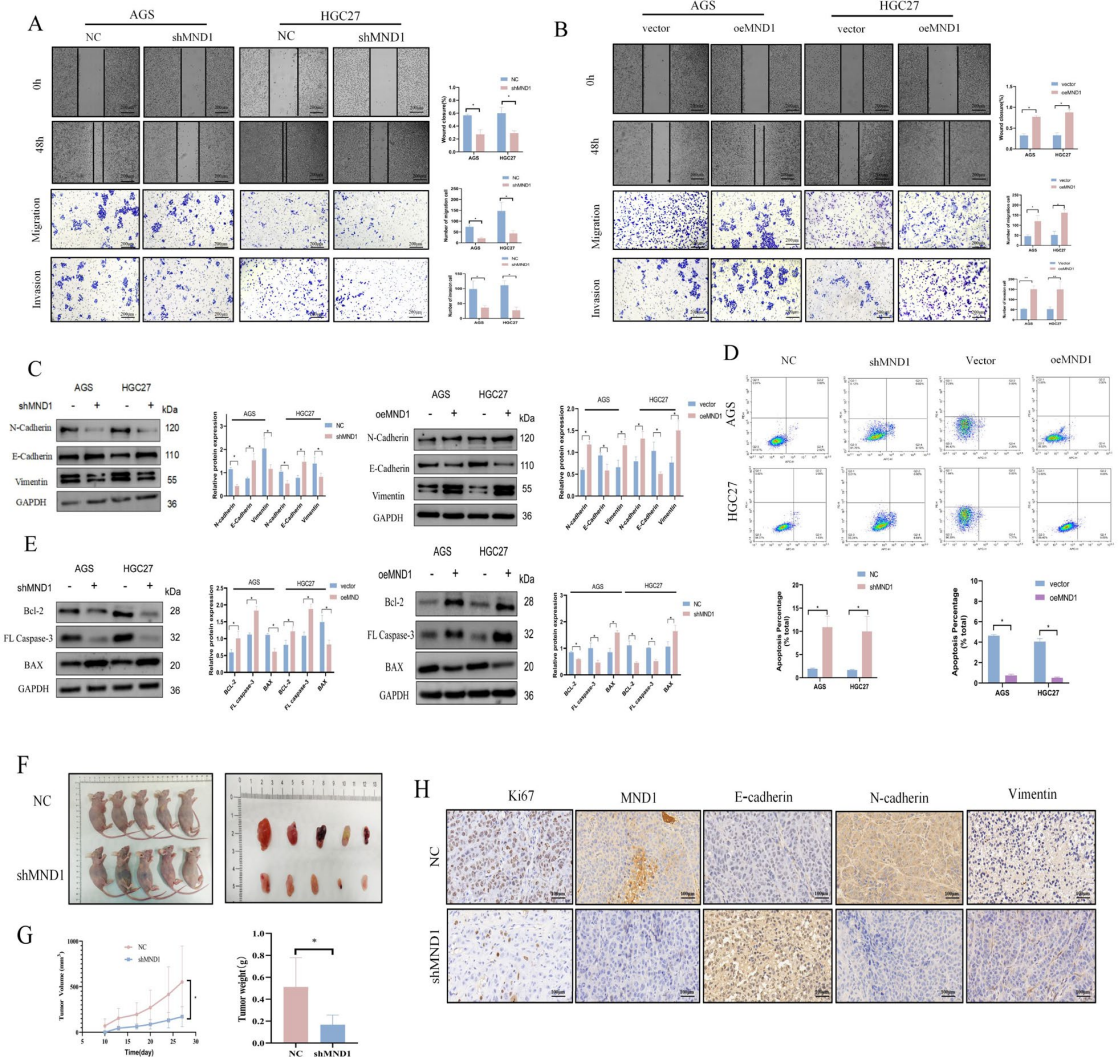
MND1 promotes invasion, migration, and apoptotic escape of GC cells. **A**, **B** Cell migration and invasion abilities were determined by wound healing and transwell assays after silencing and overexpression of MND1 in GC cells. **C** WB was used to evaluate the effects of silencing and overexpression of MND1 on EMT-related proteins. **D** Annexin V-APC/PI double staining was used to detect apoptosis after silencing and overexpression of MND1. **E** WB was used to evaluate the effects of silencing and overexpression of MND1 on apoptosis. **F** Subcutaneous tumorigenesis in nude mice was performed to evaluate the effect of MND1 silencing on tumor proliferation. **G** Tumor growth curve and tumor weight in nude mice. **H** Immunohistochemistry was used to detect the expressions of Ki67, MND1, E-cadherin, N-Carherin and Vimentin in nude mice (*P-value < 0.05, **P-value < 0.01, ***P-value < 0.001). All figures represent mean ± SD from three independent experiments

Further examination of the effect of MND1 on the ability of metastasis showed that the silencing of MND1 inhibited the migration and invasion abilities of GC cells (Fig. 3A, B). in the oeMND1 group, the transfer ability increased (Fig. 3A–B). The epithelial-mesenchymal transition (EMT) serves as a crucial role in the initiation of metastasis. WB results showed that, expressions of N-cadherin and Vimentin significantly decreased, whereas E-cadherin expression significantly increased after the silencing of MND1 (Fig. 3C). In contrast, overexpression of MND1 significantly promoted the invasion, migration, and EMT in GC cells (Fig. 3A–C).

We further explored the effect of MND1 on GC cells apoptosis. Annexin V-APC/PI results revealed that the apoptosis rate significantly increased after the silencing of MND1 (Fig. 3D). In addition, MND1 silencing can promote the expression of bcl-2 and caspase-3 and inhibit the expression of BAX. The opposite was true in the oeMND1 group (Fig. 3E). In vivo experiments further revealed that, after the silencing of MND1, the subcutaneous tumors significantly reduced and the expression of ki67 significantly decreased in the nude mice (Fig. 3F–H). IHC results showed that the expression of E-cadherin was increased, whereas the expressions of N-cadherin and Vimentin were decreased in the shMND1 group (Fig. 3H). These results suggested that MND1 promoted GC cell metastasis in vitro and in vivo.

### MND1 silencing increased the sensitivity of AGS and HGC27 cells to oxaliplatin

**Fig. 4:**
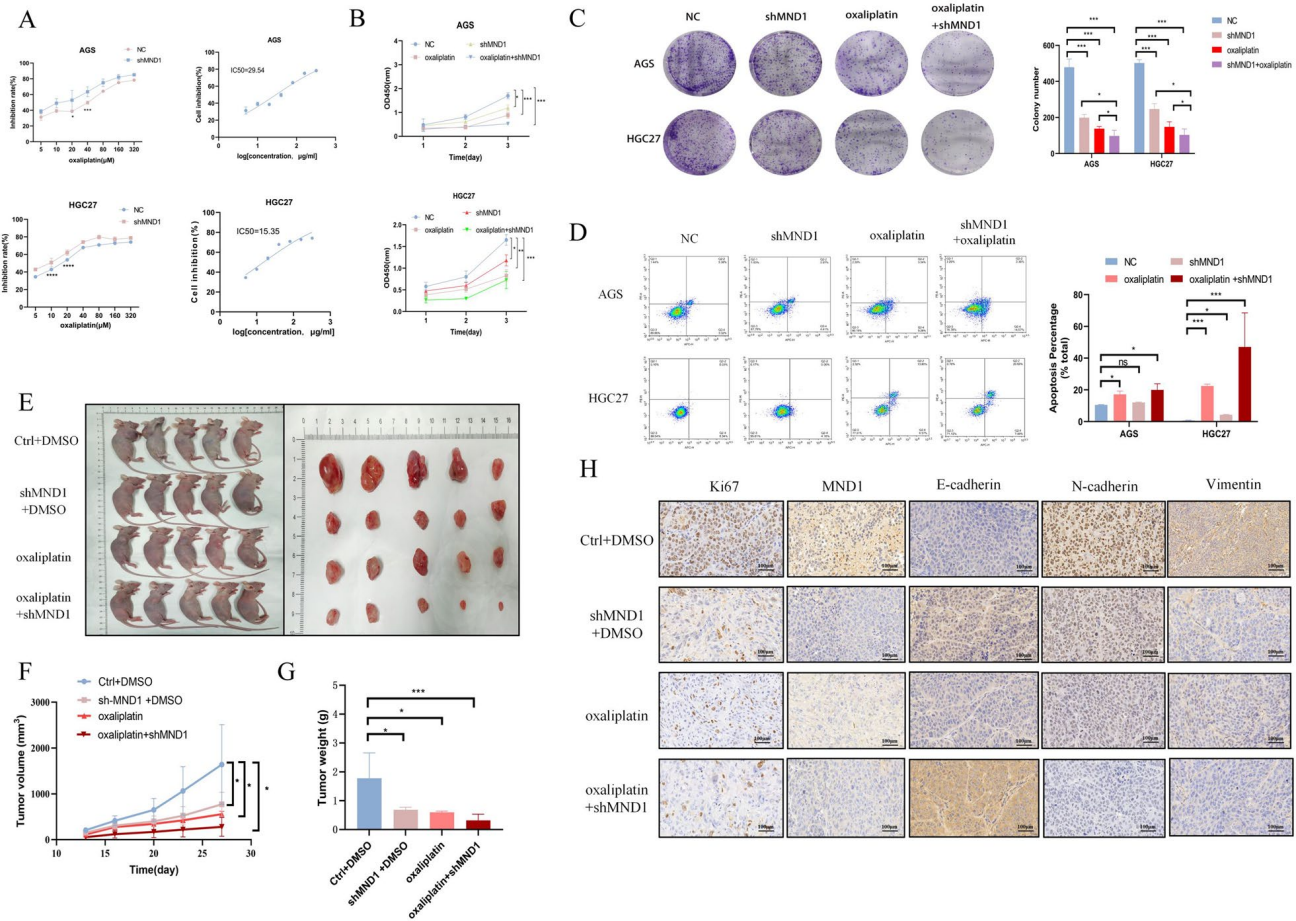
MND1 increases the sensitivity to oxaliplatin in vitro and in vivo. **A** IC50 curve and optimal dose of oxaliplatin in GC cells. **B, C** CCK-8 and colony cloning assay were used to detect proliferation in each group. **D** Annexin V-APC/PI double staining was used to detect apoptosis in each group. **E** Subcutaneous tumorigenesis was performed in nude mice in each group. **F, G** Tumor growth curve and tumor weight in nude mice. **H** The expressions of Ki67, MND1, E-cadherin, N-Carherin and Vimentin were detected by immunohistochemistry in nude mice (*P-value < 0.05, **P-value < 0.01, ***P-value < 0.001). All figures represent mean ± SD from three independent experiments

MND1 has been reported to be involved in the process of platinum resistance in lung cancer [11]. The IC50 results illustrated that the IC50 value was 29.54 for AGS and 15.35 for HGC27 (Fig. 4A). The optimal concentrations of AGS and HGC27 were found to be 40 μmol/L and 20 μmol/L respectively (Fig. 4A). Subsequently, CCK-8 and cloning experiments showed that, when compared with other groups, the proliferative capacity was most significantly decreased in the shMND1 + oxaliplatin group (Fig. 4B–C). Annexin V-APC/PI double staining showed that apoptosis rate was highest in shMND1 + oxaliplatin group compared with other groups(Fig. 4D). In vivo experiments, there was the smallest tumor size in the shMND1 + oxaliplatin group (Fig. 4 E–G). IHC results indicated that expressions of ki67, MND1, N-cadherin, and Vimentin were decreased, whereas E-cadherin was elevated in the shMND1 + oxaliplatin group (Fig. 4H). The results suggested that MND1 was involved in the sensitivity of GC cells to oxaliplatin.

### FOXA1 binds to the MND1 promoter and inhibits GC cell proliferation, invasion, migration, and apoptotic escape

**Fig. 5:**
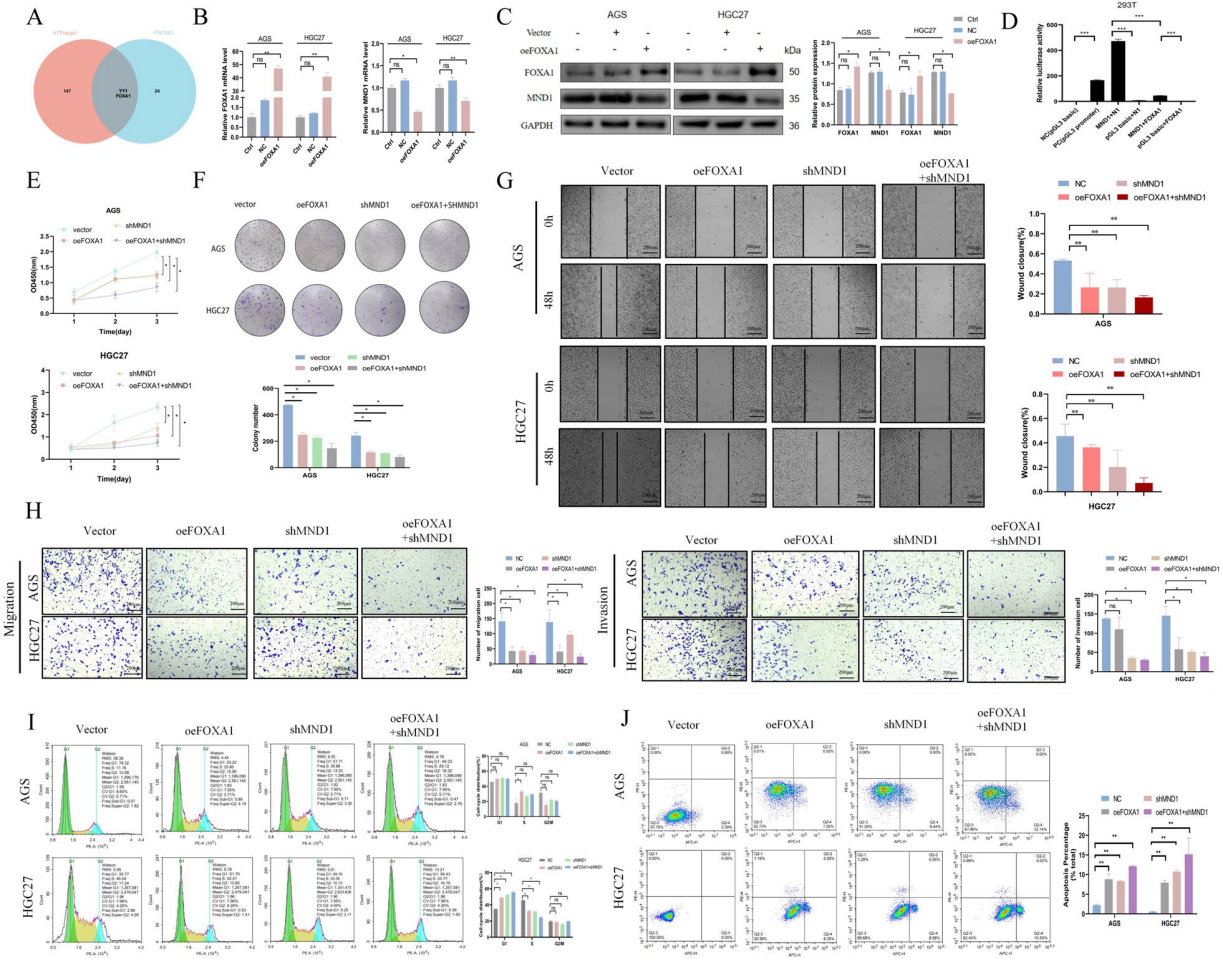
FOXA1 binds to the MND1 promoter and affects the progression of GC cells. A. Venn diagrams based on the hTFtarget and PROMO databases predicted the transcription factors bound to the MND1 promoter. **B, C** QRT-PCR and WB were used to detect the expressions of FOXA1 and MND1 after overexpression of FOXA1. **D** The double luciferase reporter gene assay showed the direct binding of FOXA1 to MND1. **E, F** CCK-8 and clone colony test were used to detect the proliferation in each group. **G, H** Cell migration and invasion abilities in each group was detected by wound healing and transwell assays. **I** Flow cytometry was used to detect the changes of cell cycle in each group. **J** Annexin V-APC/PI double staining was used to detect apoptosis in each group (*P-value < 0.05, **P-value < 0.01, ***P-value < 0.001). All figures represent mean ± SD from three independent experiments

Transcription factors bound to the MND1 promoter were further predicted using the hTFtarget (n = 412) and PROMO databases (n = 25). The Venn diagram identified two common genes (YY1 and FOXA1, Fig. 5 A and Additional file 9: Table S8). Subsequently, PCR and WB results showed that the level of MND1 significantly decreased after overexpression of FOXA1 (Fig. 5B, C). The results of dual luciferase indicated that the fluorescence intensity of MND1 + FOXA1 group was lower than that of MND1 + N1 group. This suggests that FOXA1 binds to MND1 and inhibits its transcription process (Fig. 5D). The results of CCK-8, cloning, wound healing, migration and invasion experiments showed that, compared with the Vector group, the proliferation and metastasis ability were decreased in oeFOXA1, shMND1 and oeFOXA1 + shMND1 groups, especially in oeFOXA1 + shMND1 group (Fig. 5E–H). In addition, oeFOXA1 + shMND1 group showed the most significant increase in G1 phase cell cycle arrest (Fig. 5I) and significantly increased apoptosis rate (Fig. 5 J). These results indicated that FOXA1 could bind to the promoter of MND1, regulate mRNA level of MND1, and inhibit GC cell proliferation, metastasis, and apoptosis escape.

### MND1 can directly bind to TKT and regulate GC cell proliferation, metastasis, and glucose metabolism via the PI3K/AKT signaling pathway

**Fig. 6:**
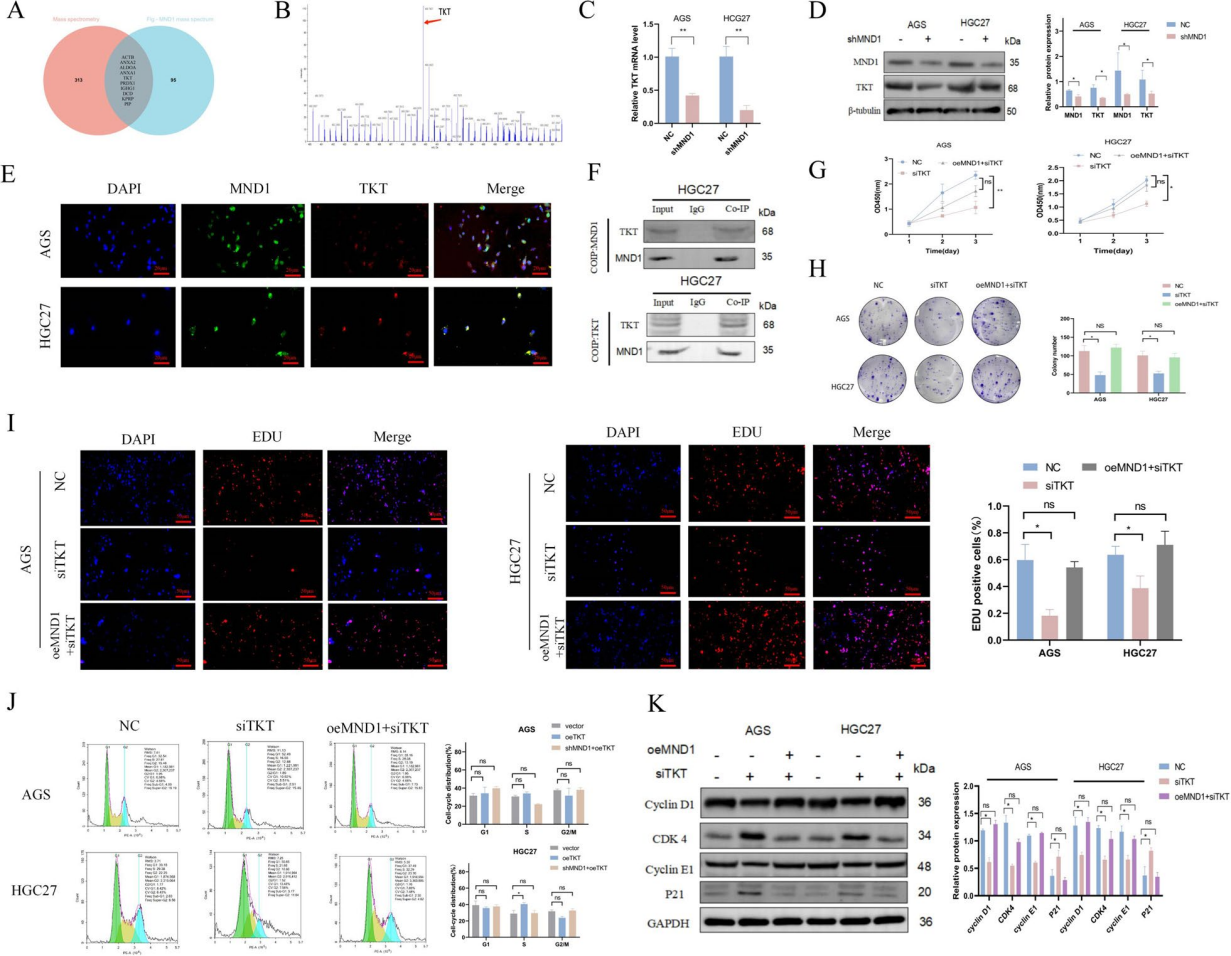
MND1 directly binds to TKT and regulates proliferation of GC cells. **A** The intersection genes were detected by Venn diagram based on mass spectrometry and flg-MND1 mass spectrum. **B** Peak plot of TKT in mass spectrometry. **C, D** QRT-PCR and WB were used to detect the expression of TKT after MND1 silencing. **E** MND1 and TKT localizations were examined by immunofluorescence. **F** CO-IP was used to detect the combination of TKT and MND1. **G, H** CCK-8 and cell cloning assay were used to detect the proliferation after MND1 silencing. **I** EDU was used to detect the proliferation after MND1 silencing. **J** Flow cytometry was used to detect the changes of cell cycle in each group. **K** Cell cycle-related proteins were detected by WB (*P-value < 0.05, **P-value < 0.01, ***P-value < 0.001). All figures represent mean ± SD from three independent experiments. 83 84 85

The potential molecular mechanism and candidate proteins interacting with MND1 were further studied. Venn diagram based on mass spectrometry and Flg-MND1 mass spectrum identified 10 potential genes (Fig. 6A and Additional file 10: Table S9), and the gene with the highest binding degree (TKT, Fig. 6B) was selected for further verification. We founded that the expression of TKT decreased after the silencing of MND1 (Fig. 6C, D). The immunofluorescence further demonstrated that MND1 and TKT were colocalized in the cytoplasm and nucleus (Fig. 6E). The interaction between MND1 and TKT was further validated by Co-IP (Fig. 6F).

**Fig. 7:**
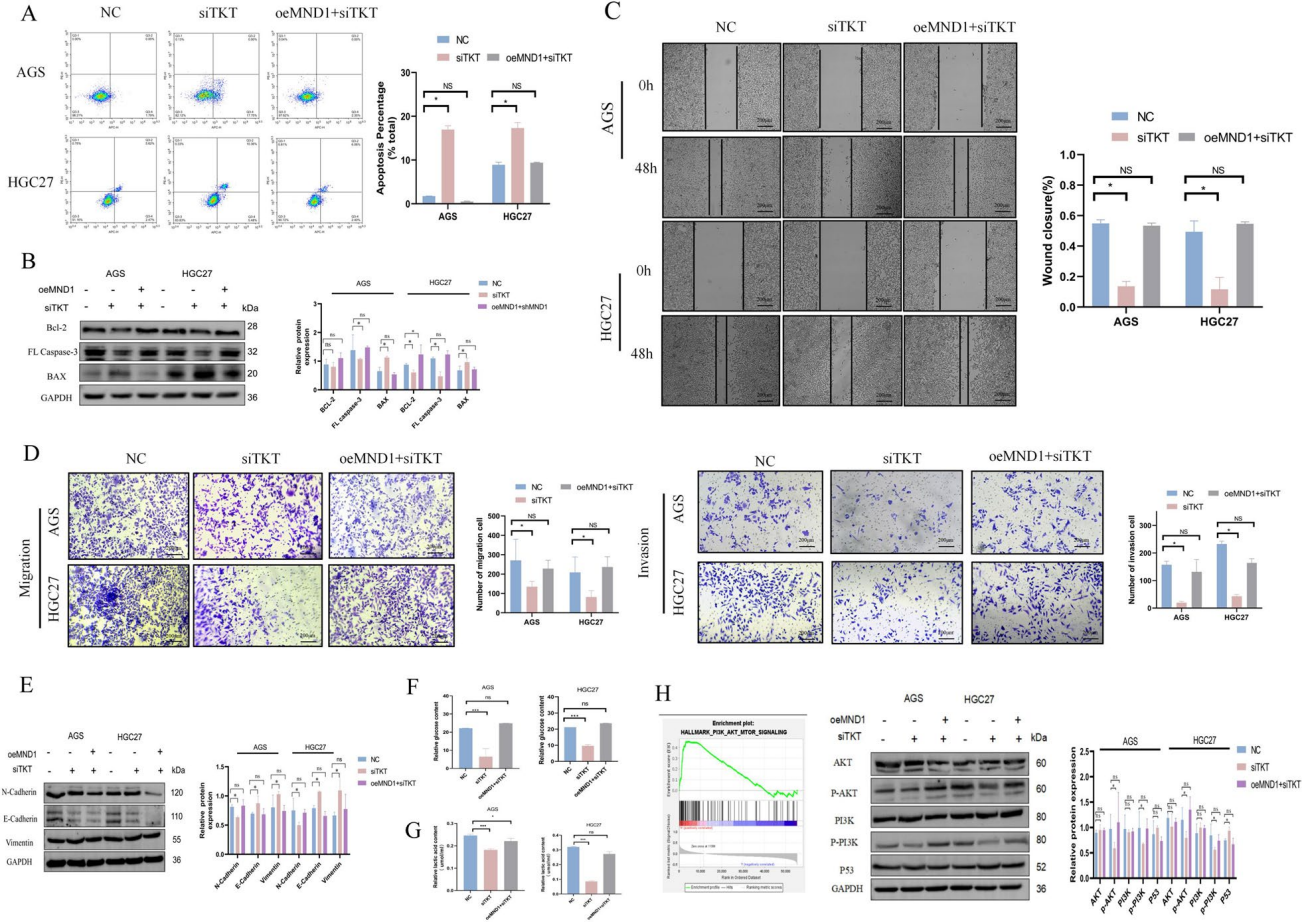
TKT silencing inhibits metastasis and reduces lactic acid production and glucose uptake in GC cells. **A, B** Annexin V-APC/PI double staining and WB were used to detect apoptosis. **C, D** Wound healing assay and transwell assay were used to detect cell migration and invasion abilities. **E** EMT related proteins were detected by WB. **F** Lactic acid production was detected by the L-lactic acid detection kit. **G** The glucose uptake assay was performed by the glucose detection kit. **H** The TKT enrichment signaling pathway was detected by GSEA and the expressions of AKT, P-Akt, PI3K, P-PI3K and P53 were detected by WB (*P-value < 0.05, **P-value < 0.01, ***P-value < 0.001). All figures represent mean ± SD from three independent experiments

In addition, the silence of TKT reduced GC cell proliferation (Fig. 6 G-I), increased cell cycle arrest in G1 phase (Fig. 6 J, K), promoted GC cell apoptosis (Fig. 7A, B), decreased metastasis (Fig. 7C–E) and total glucose and lactate uptake capacity (Fig. 7F–G). The enrichment analysis for GSEA pathway identified the PI3K/AKT signaling pathway as a downstream target of TKT. Further verification by WB showed that, after silencing TKT, the levels of P-AKT and p-PI3K decreased, while the level of P53 increased, which could be rescued by the overexpression of MND1 (Fig. 7H).

**Fig. 8:**
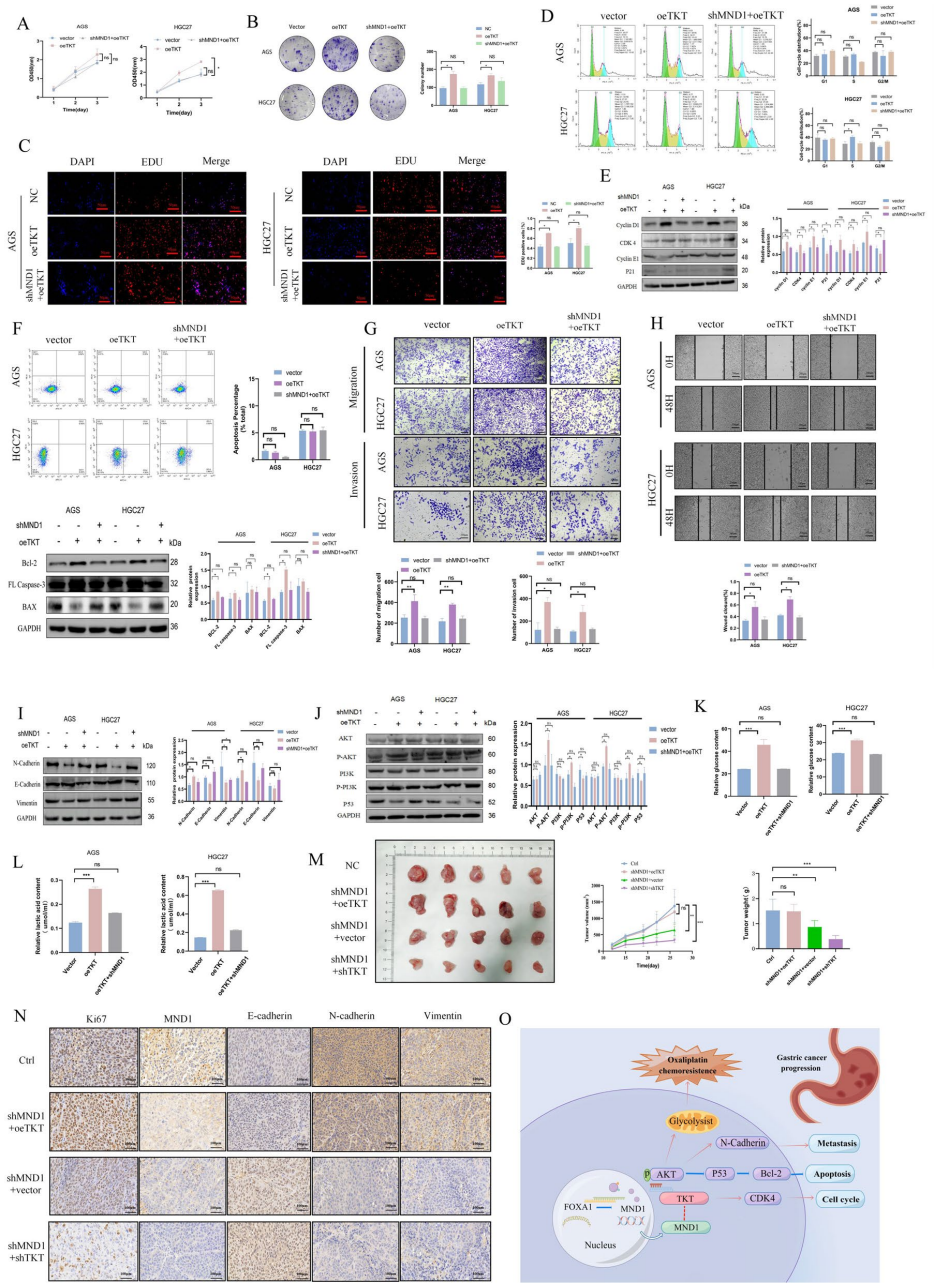
Overexpression of TKT promotes the proliferation, metastasis, lactic acid and glucose uptake in GC cells. **A, B** The proliferation was detected by CCK-8 and clone assay in each group. **C** The proliferation was detected by EDU in each group. **D** The changes of cell cycle were detected by flow cytometry in each group. **E** The changes of cycle-critical proteins were detected by WB. **F** Annexin V-APC/PI and WB were used to detect apoptosis and apoptosis-related proteins. **G, H** The cell migration and invasion abilities were detected by wound healing and transwell assays. **I, J** Changes of EMT and PI3K/AKT signaling pathway related proteins were detected by WB. **K, L** Changes of lactic acid and total glucose in each group. **M** Subcutaneous tumorigenesis was performed in nude mice in each group. **N** The expressions of Ki67, MND1, E-cadherin, N-cadherin and Vimentin were detected by IHC. **O** Schematic diagram of the role of FOXA1/MND1/TKT signaling axis in regulating GC progression and oxaliplatin sensitivity via PI3K/AKT signaling pathway (*P-value < 0.05, **P-value < 0.01, ***P-value < 0.001). All figures represent mean ± SD from three independent experiments

To further verify that TKT interferes with GC cell progression, we constructed plasmids overexpressing TKT. Overexpression of TKT promoted GC cell proliferation (Fig. 8A–C), drived cell cycle progression (Fig. 8D–E), decreased early apoptosis (Fig. 8F), promoted cell migration (Fig. 8G–I), and increased total glucose and lactic acid uptake (Fig. 8 K–L). Subsequently, nude mice were randomly divided into NC, shMND1 + oeTKT, shMND1 + vector, and shMND1 + shTKT groups to further assess the effect of TKT on proliferation. In the shMND1 + shTKT group, tumor volume was the smallest, the expressions of ki67, MND1, N-cadherin, and Vimentin were the lowest, and the expression of E-cadherin was the highest (Fig. 8 M–N). These results demonstrated that TKT was involved in regulating the proliferation, invasion, migration, and glycolytic metabolism of GC cells.

## Discussion

Cancer can regulate the activity of genes by activating meiotic chromosomes, leading to deviations in the mechanisms that control chromosome segregation and maintenance. MND1 is directly related to poor prognosis in breast cancer and lung adenocarcinoma patients [18, 19]. In the present study, the high expression of MND1 was found to be associated with an advanced pathological grade and TNM stage, and was an adverse prognosis factor in patients with GC. Previous literature has documented that cell cycle-related proteins are associated with platinum resistance [20]. Moreover, CDK4/6 inhibitors promote the efficacy of oxaliplatin [21]. In this study, silencing MND1 increased the sensitivity of the GC cells to oxaliplatin and supplyment with oxaliplatin after silencing MND1 promoted the GC cells apoptosis. This finding is consistent with the report by Zhang et al., who demonstrated that MND1 competitively bound to KLF6 and conferred cisplatin resistance in lung cancer [10]. MND1 may be involved in cyclin regulation, thereby controlling the process of oxaliplatin resistance.

FOXA1 is a member of the FOX family. As a transcription factor, FOXA1 is involved in the biological processes of various cancers [22]. FOXA1 can be degraded via ubiquitination of ZFP91 in GC to promote the cell progression [23]. Meanwhile, FOXA1 can inhibit the proliferation, EMT, and apoptotic escape of GC cells [24]. FOXA1 has a highly-conserved winged-helix DNA-binding domain that can replace histone H1 and maintain the open conformation of the enhancer region, thereby activating the target gene expression [25, 26]. This study proposes for the first time that FOXA1 binds to the promoter region of MND1 and represses the MND1 expression. After the combination of FOXA1 overexpression and MND1 silencing, the inhibitory effect on the proliferation of GC cells is more evident, further indicating that FOXA1 inhibits the expression of MND1 and thereby induces a biological effect.

Cancer cells induce metastasis and chemoresistance via increased glucose uptake and the creation of a more acidic tumor microenvironment [27]. By using MS and CO-IP, this study revealed, for the first time, that MND1 can be coexpressed with TKT. TKT is mainly involved in de novo nucleotide synthesis and pentose phosphate pathway toward the promotion of rapid cancer cell proliferation [28]. Furthermore, TKT can enhance oxidative stress as well as the proliferative and metastatic capacities of hepatoma cells in vitro and promote tumor progression by binding to the EGFR pathway [29]. Our results showed that silencing TKT could promote the proliferation of GC cells, which is consistent with past report [30]. Tumor metastasis is inseparable from the enhancement of glycolytic capacity [31]. We also found that silence of TKT can inhibit metastasis and reduce lactate generation and glucose uptake in GC cells. We speculate that TKT promotes the process of glycolysis and maintains the acidic tumor microenvironment, which facilitates metastasis of GC cells.

To date, the mechanism of TKT involved in oxaliplatin resistance is not clear. It has been reported that TKT activates the AKT expression via GRP78 phosphorylation [30]. Moreover, AKT can phosphorylate TKT at thr382 to promote its expression and purine synthesis [32]. Interestingly, TKT was significantly overexpressed in samples of oxaliplatin resistant colorectal cancer patients [32]. It may be that TKT affects the aerobic glycolysis process of the tumor,,which is involved in oxaliplatin resistance. Park SY et al. [33] recently found that inhibition of AMPK in oxaliplatin-resistant colorectal cancer cells induced autophagy by silencing AKT/mTOR pathway and reducing glycolytic enzymes. Therefore, targeting AMPK may increase the sensitivity of colorectal cancer cells to oxaliplatin. Overexpression of MiR-138 suppressed the PDK1 expression to decrease the oxaliplatin resistance of colorectal cancerr [34]. Cheng et al. demonstrated that the down-regulation of *PTBP1* gene can overcome oxaliplatin resistance of drug-resistant colon cancer cells by regulating glycolysis [35]. In addition, the PI3K/AKT signaling pathway has also been reported to be involved in mediating glycolysis and promoting oxaliplatin resistance. Fang et al. has recently shows that AKT mediated phosphorylation of *TOPBP1* at Ser1159 is involved in the activation of oxaliplatin resistant GC cells [36]. In colon cancer cells, *SHP2* activates AKT to promote oxaliplatin resistance [37].Our study found that TKT activated the phosphorylated expression of AKT and oxaliplatin resistance process of GC cells, which are consistent with the above reports [33–37]. As a whole, this study indicating that TKT activates AKT and exerts functional effects via the PI3K/AKT signaling axis (Fig. 8O).

Tumor cells can obtain energy via glycolysis and pentose phosphate pathways, thereby enhancing their resistance to chemotherapeutic drugs [11, 38]. Our study found that MND1 attenuated the toxic effects of oxaliplatin on the GC cells. Furthermore, our findings were validated in a nude mouse model. However, past studies have demonstrated that TKT is overexpressed in patients receiving oxaliplatin chemotherapy, which may be related to the activation of the glucose metabolism pathways after the tumor cells are damaged [39]. Unfortunately, the correlation between TKT and oxaliplatin resistance is out of the scope of this study, and further investigation is needed.

## Conclusion

In conclusion, this study elucidated the effect of the FOXA1/MND1/TKT axis on the GC cell progression and oxaliplatin sensitivity via the PI3K/AKT signaling pathway. These findings are expected to contribute to the understanding of oxaliplatin resistance and molecular mechanisms in GC cells.

### Supplementary Information

**Additional file 1: Figure S1.** MND1 expression and subgroup survival analysis in various cancers. **A** Intersection of differential genes of TCGA, GES70880 and GSE99416; **B** Intersection of differential genes and DDP- drug resistance (GSE122130)l; **C** Based on the TCGA database, the expression of MND1 in various cancer types was detected（Green is the adjacent tissue and red is the cancerous tissue）; **D** OS prognosis of different MND1 expression in pathological stage and TNM stage in 159 GC patients. **Figure S2.** IHC staining of MND1 in cancer and adjacent normal cancer based on tissue microarray.

**Additional file 2: Table S1.** Up-regulated gene sets of TCGA, GSE70880 and GSE99416.

**Additional file 3: Table S2.** Gene sets of DEGS and GSE122130.

**Additional file 4: Table S3.** Primers sequences used in this work.

**Additional file 5: Table 4.** Antibodies used in this work.

**Additional file 6: Table 5.** Clinical information on 159 patients in THE TME chip.

**Additional file 7: Table 6.** Characteristic data table of 159 patients.

**Additional file 8: Table 7.** Correlation between characteristics and OS of GC patients.

**Additional file 9: Table 8.** HTFtarget database and PROMO database intersection results.

**Additional file 10: Table 9.** Mass spectrometry and Flg-MND1 mass spectrum results.

## Data Availability

The datasets used and analyzed during the current study are available from the corresponding author on reasonable request.

## Abbreviations

MND1: Meiotic nuclear division 1
GC: Gastric cancer
FOXA1: Forkhead box protein A1
MS: Mass spectrometry
CO-IP: Co-immunoprecipitation
TKT: Transketolase
TCGA: The cancer genome atlas
GEPIA: Gene expression profiling interactive analysis
TMA: Tissue microarray
IHC: Immunohistochemistry
IF: Immunofluorescence
