# Supplementary material for: FOXA1/MND1/TKT axis regulates gastric cancer progression and oxaliplatin sensitivity via PI3K/AKT signaling pathway
Source: Cancer Cell Int. 2023 Oct 10;23:234. doi: 10.1186/s12935-023-03077-4 (PMC10566187; doi:10.1186/s12935-023-03077-4)
Supplement: Supplementary file 1 — Additional file 1: Figure S1. MND1 expression and subgroup survival analysis in various cancers. A Intersection of differential genes of TCGA, GES70880 and GSE99416; B Intersection of differential genes and DDP- drug resistance (GSE122130)l; C Based on the TCGA database, the expression of MND1 in various cancer types was detected（Green is the adjacent tissue and red is the cancerous tissue）; D OS prognosis of different MND1 expression in pathological stage and TNM stage in 159 GC patients. Figure S2. IHC staining of MND1 in cancer and adjacent normal cancer based on tissue microarray. [file 12935_2023_3077_MOESM1_ESM.pdf]

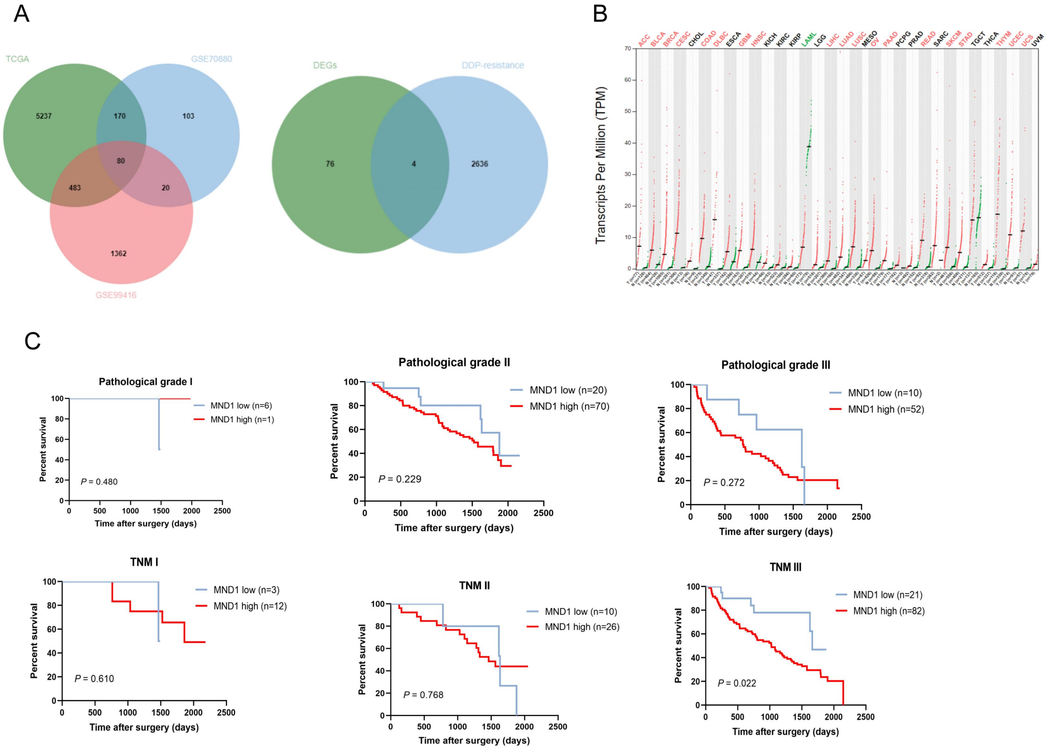

Supplementary Figure 1. MND1 expression and subgroup survival analysis in various cancers. **A**.Intersection of differential genes of TCGA, GES70880 and GSE99416 on the left. Intersection of differential genes and DDP- drug resistance (GSE122130) on the right; **B**.Based on the TCGA database, the expression of MND1 in various cancer types was detected（Green is the adjacent tissue and red is the cancerous tissue）; **C**. OS prognosis of different MND1 expression in pathological stage and TNM stage in 159 GC patients.

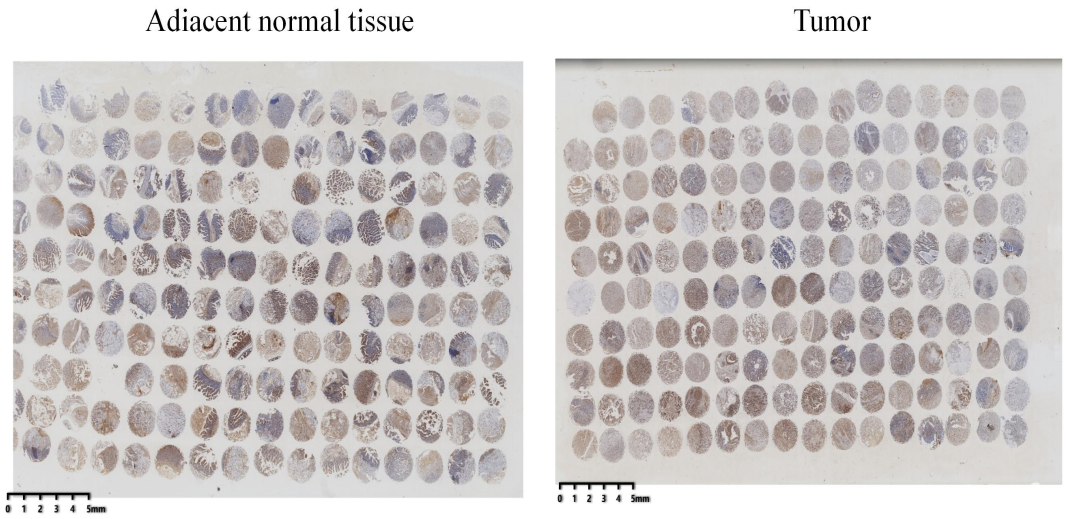

Supplementary Figure 2. IHC staining of MND1 in cancer and adjacent normal cancer based on tissue microarray.
