# Supplementary material for: FOXA1/MND1/TKT axis regulates gastric cancer progression and oxaliplatin sensitivity via PI3K/AKT signaling pathway
Source: Cancer Cell Int. 2023 Oct 10;23:234. doi: 10.1186/s12935-023-03077-4 (PMC10566187; doi:10.1186/s12935-023-03077-4)
Supplement: Supplementary file 4 — Additional file 4: Table S3. Primers sequences used in this work. [file 12935_2023_3077_MOESM4_ESM.doc]

Supplementary Table 3 Primers sequences used in this work

| Gene | Forward Primer (5'-3') | Reverse Primer (5'-3') |
| --- | --- | --- |
| MND1 | CAGTGCCGAAGAATCGGTCAT | CGAGCAGCATTTAATGGAGACA |
| TKT | TCCACACCATGCGCTACAAG | CAAGTCGGAGCTGATCTTCCT |
| FOXA1 | GCAATACTCGCCTTACGGCT | TACACACCTTGGTAGTACGCC |
