# Supplementary material for: FOXA1/MND1/TKT axis regulates gastric cancer progression and oxaliplatin sensitivity via PI3K/AKT signaling pathway
Source: Cancer Cell Int. 2023 Oct 10;23:234. doi: 10.1186/s12935-023-03077-4 (PMC10566187; doi:10.1186/s12935-023-03077-4)
Supplement: Supplementary file 5 — Additional file 5: Table 4. Antibodies used in this work. [file 12935_2023_3077_MOESM5_ESM.doc]

Supplementary Table 4 Antibodies used in this work

| Antibodies | Manufacturer | Number | Usage |
| --- | --- | --- | --- |
| MND1 | Proteintech | 11636-1-AP | WB, IF, IHC, Co-IP, |
| FOXA1 | Abcam | ab170933 | WB |
| TKT | Proteintech | 11039-1-AP | WB,IF |
| CDK4 | Proteintech | 11026-1-AP | WB |
| Cyclin D1 | Proteintech | 26939-1-AP | WB |
| Cyclin E1 | Proteintech | 11554-1-AP | WB |
| Bcl-2 | Abcam | ab32124 | WB |
| BAX | Abcam | ab182733 | WB |
| Caspase-3 | Abcam | ab32351 | WB |
| Vimentin | Proteintech | 10366-1-AP | WB,IHC |
| E-Cadherin | Proteintech | 20874-1-AP | WB,IHC |
| N-Cadherin | Proteintech | 22018-1-AP | WB,IHC |
| AKT | Proteintech | 60203-2-Ig | WB |
| P-AKT | Proteintech | 66444-1-Ig | WB |
| PI3K | Proteintech | 20584-1-AP | WB |
| P-PI3K | Abcam | ab140307 | WB |
| P53 | Abcam | ab26 | WB |
| P21 | Proteintech | 10355-1-AP | WB |
| Normal rabbit IgG | Cell Signaling Technology | #2729 | CO-IP |
| Normal mouse IgG | Santa Cruz Biotechnology | sc-2025 | CO-IP |
