# Supplementary material for: FOXA1/MND1/TKT axis regulates gastric cancer progression and oxaliplatin sensitivity via PI3K/AKT signaling pathway
Source: Cancer Cell Int. 2023 Oct 10;23:234. doi: 10.1186/s12935-023-03077-4 (PMC10566187; doi:10.1186/s12935-023-03077-4)
Supplement: Supplementary file 7 — Additional file 7: Table 6. Characteristic data table of 159 patients. [file 12935_2023_3077_MOESM7_ESM.doc]

| **Variables** | **Overall(n=159)** | **MND1 level** | | ***P* value** |
| --- | --- | --- | --- | --- |
|  |  | **low(n=36)** | **high(n=123)** |  |
| Age（years） |  |  |  | 0.576 |
| ＜65 | 73 | 18 | 55 |  |
| ≥65 | 86 | 18 | 68 |  |
| Tumor grade |  |  |  | **＜0.001** |
| Ⅰ | 7 | 6 | 1 |  |
| Ⅱ | 90 | 20 | 70 |  |
| Ⅲ | 62 | 10 | 52 |  |
| Tumor size |  |  |  | 0.764 |
| ＜5cm | 83 | 18 | 65 |  |
| ≥5cm | 76 | 18 | 58 |  |
| Nerve invasion |  |  |  | **0.004** |
| yes | 31 | 1 | 30 |  |
| no | 128 | 35 | 93 |  |
| Vessel carcinoma embolus |  |  |  | 0.672 |
| yes | 35 | 7 | 28 |  |
| no | 124 | 29 | 95 |  |
| Number of lymph node metastasis |  |  |  | 0.898 |
| ＜3 | 78 | 18 | 60 |  |
| ≥3 | 81 | 18 | 63 |  |
| T stage |  |  |  | **＜0.001** |
| T1 | 9 | 6 | 3 |  |
| T2 | 32 | 18 | 14 |  |
| T3 | 19 | 6 | 13 |  |
| T4 | 99 | 6 | 93 |  |
| N stage |  |  |  | 0.780 |
| N0 | 44 | 12 | 32 |  |
| N1 | 34 | 6 | 28 |  |
| N2 | 34 | 8 | 26 |  |
| N3 | 47 | 10 | 37 |  |
| M stage |  |  |  | 0.690 |
| M0 | 154 | 34 | 120 |  |
| M1 | 5 | 2 | 3 |  |

Supplementary Table 6 Characteristic data table of 159 patients
