# Supplementary material for: FOXA1/MND1/TKT axis regulates gastric cancer progression and oxaliplatin sensitivity via PI3K/AKT signaling pathway
Source: Cancer Cell Int. 2023 Oct 10;23:234. doi: 10.1186/s12935-023-03077-4 (PMC10566187; doi:10.1186/s12935-023-03077-4)
Supplement: Supplementary file 8 — Additional file 8: Table 7. Correlation between characteristics and OS of GC patients. [file 12935_2023_3077_MOESM8_ESM.doc]

Supplementary Table 7. Correlation between characteristics and OS of GC patients.

| **Variables** | **Univariate analysis** | | **Multivariate analysis** | |
| --- | --- | --- | --- | --- |
|  | **HR (95% CI)** | ***P* value** | **HR (95% CI)** | ***P* value** |
| Age (≥65 *vs.* <65 ys) | 1.014(0.674-1.524) | 0.947 |  |  |
| Tumor grade (Ⅱ+Ⅲ *vs.* Ⅰ) | 4.458(0.621-31.994) | 0.137 |  |  |
| Tumor size (≥5 *vs.* <5 cm) | 1.177(0.786-1.761) | 0.429 |  |  |
| Nerve invasion (yes *vs.* no) | 1.480(0.917-2.388) | 0.108 |  |  |
| Number of lymph node metastasis  (≥3 *vs.* <3) | 1.629(1.083-2.461) | **0.004** | 1.688(1.121-2.543) | **0.012** |
| TNM stage (III-IV *vs.* I-II) | 1.842(1.158-2.930) | **0.010** | 1.887(1.186-3.004) | **0.007** |
| T stage (T2-T4 *vs.* T1) | 3.465(0.853-14.071) | 0.082 |  |  |
| N stage (N1-N4 *vs.* N0) | 2.035(1.237-3.346) | **0.005** | 2.080(1.261-3.430) | **0.004** |
| M stage (M1 *vs.* M0) | 1.734(0.626-4.805) | 0.114 |  |  |
| MND1 level (high *vs.* low) | 1.848(1.008-3.388) | **0.047** | 1.916(1.045-3.513) | **0.036** |

Abbreviations: HR, hazardous radio.
